# Thriving from Work: Conceptualization and Measurement

**DOI:** 10.3390/ijerph18137196

**Published:** 2021-07-05

**Authors:** Susan E. Peters, Glorian Sorensen, Jeffrey N. Katz, Daniel A. Gundersen, Gregory R. Wagner

**Affiliations:** 1 Department of Social and Behavioral Sciences, Harvard T.H. Chan School of Public Health, Boston, MA 02115, USA; glorian_sorensen@dfci.harvard.edu; 2 Dana-Farber Cancer Institute, Boston, MA 02215, USA; 3 Departments of Orthopedic Surgery and Medicine, Brigham and Women’s Hospital, Boston, MA 02115, USA; jnkatz@bwh.harvard.edu; 4 Department of Epidemiology, Harvard T.H. Chan School of Public Health, Boston, MA 02115, USA; 5 Survey and Qualitative Methods Core, Division of Population Health, Dana-Farber Cancer Institute, Boston, MA 02215, USA; DanielA_Gundersen@dfci.harvard.edu; 6 Department of Environmental Health, Harvard T.H. Chan School of Public Health, Boston, MA 02115, USA; grwagner@post.harvard.edu

**Keywords:** total worker health, healthy work design and well-being, flourishing, questionnaire design, survey design, cognitive interviewing, measurement of well-being, thriving, worker well-being, worker thriving

## Abstract

Work is a major contributor to our health and well-being. Workers’ thriving is directly influenced by their job design, work environment and organization. The purpose of this report is to describe the qualitative methods used to develop the candidate items for a novel measure of Thriving from Work through a multi-step iterative process including: a literature review, workshop, interviews with experts, and cognitive testing of the candidate items. Through this process, we defined Thriving from Work as the state of positive mental, physical, and social functioning in which workers’ experiences of their work and working conditions enable them to thrive in their overall lives, contributing to their ability to achieve their full potential in their work, home, and community. Thriving from Work was conceptualized into 37 attributes across seven dimensions: psychological, emotional, social, work–life integration, basic needs, experience of work, and health. We ultimately identified, developed and/or modified 87 candidate questionnaire items mapped to these attributes that performed well in cognitive testing in demographically and occupationally diverse workers. The Thriving from Work Questionnaire will be subjected to psychometric testing and item reduction in future studies. Individual items demonstrated face validity and good cognitive response properties and may be used independently from the questionnaire.

## 1. Introduction

Work is a central human life function, with workers, on average, spending over one-third of their waking hours performing work-related activities [1]. Work plays a major role in shaping health and well-being; thus, employment is a well-recognized social determinant of health and a leading health indicator for the United States’ Healthy People 2030 initiative [2,3]. Exposures to specific conditions of work—physical, organizational, and psychosocial—not only contribute to injury and increase the risk of ill-health and mental distress but may also be instrumental in fostering well-being [4]. Many people work long hours, experience work overload, have excessive emotional and cognitive job demands, and are exposed to hazardous and stressful working conditions [5]. With current technologies and in response to the COVID-19 pandemic, the lines between work and non-work commitments are blurring. Burnout [6,7,8,9,10,11,12] and substance use, such as alcohol dependence and opioid misuse [13,14,15], are on the increase in many industries. All of these circumstances are likely to have detrimental impacts on health and well-being. Positive working conditions have been associated with fostering worker well-being, such as supervisor support and leadership behaviors [16,17]; coworker support [18]; decision latitude, job and schedule control and autonomy [19,20,21]; positive organizational culture [22]; opportunities for advancement [23]; and fair pay [23].

Traditionally, occupational health and safety research has focused primarily on understanding the causes of disease and on developing strategies for limiting or mitigating risk. An expanded focus on the potential beneficial influence of work on health and well-being can provide a complementary expansion to the traditional occupational health and safety paradigm [24]. The World Health Organization’s definition of Health as *“a state of complete physical, mental and social well-being and not merely the absence of disease or infirmity”* [25], is supportive of considering both the health risks and health benefits of work.

The National Institute for Occupational Safety and Health (NIOSH) and other leading international health agencies have recognized that work can have a profound positive influence on health and well-being [26,27,28,29]. Nevertheless, this important area of research remains largely understudied. One reason for this is the lack of reliable and valid instruments that can be used in various populations to measure worker well-being in the context of the conditions of work.

Thriving contributes to an individuals’ vitality, personal and professional growth, and life satisfaction [30,31,32,33,34]. Brown et al. defined *thriving as* “*the state of positive functioning at its fullest range—mentally, physically, and socially*.” [30] (p. 256). Spreitzer et al. conceptualized a narrower view of thriving than Brown et al., centered only on vitality and learning [33,35,36]. Based on this narrower conceptualization of thriving, Porath et al. developed the ‘Thriving at Work Scale’ which focused only on these two dimensions [37], without recognition of the bidirectional nature that work and life outside work have on each other. Although this instrument has strong theoretical underpinnings, it has been critiqued based on its limited focus and interpretation; and others, such as Brown et al. [30], have recommended that a broader instrument of thriving from work is needed.

A method to assess worker’s thriving is needed in the occupational health field for several reasons. A standard measure of thriving from work would allow comparisons across worker populations and across studies of the same population, and could be used to evaluate the effect of interventions on a common scale. Our goal is to design an instrument that can be used for both research and practice with diverse applications: (a) as an outcome measure of workers’ thriving, and dimensions of workers’ thriving, (b) as a vehicle for periodic surveillance across a diversely employed worker population or within a single employing organization, (c) as an organizational diagnostic tool to identify priority areas for interventions to improve worker well-being, and (d) as a means to measure or monitor intervention, policy, or program effectiveness. Thriving from work is an important, complex, worker well-being construct, that draws from both the positive psychology and occupational health literatures and encapsulates how the experience of work, and its conditions, shape a worker’s functioning and life satisfaction—not only at work but also in a worker’s life outside of work.

While the importance of developing worker well-being metrics is receiving increasing attention, there is limited literature on the topic of thriving from work; and efforts to measure both worker well-being and thriving from work are scarce. Other instruments that measure worker well-being have been developed; however, these instruments also have limitations. NIOSH’s Worker Well-being Questionnaire (WellBQ) was designed as a battery of questions mapped to a framework to examine the multiple facets of well-being, primarily to identify areas for intervention to improve worker well-being or for surveillance of these different facets [38]. However, it does not provide a single unidimensional measure of worker well-being. Some instruments have focused exclusively on the affective component of well-being, that is, how employees feel about various aspects of their work [39]. Questionnaires such as ‘Work-related Well-being Index’ [40] and ‘Work Well-being Questionnaire’ [41], focus mainly on subjective impressions of work, with only a narrow focus on the workers’ experiences of work, e.g., social well-being; and, Zheng et al.’s employee well-being scale focuses on broader aspects such as life well-being and psychological well-being not related to work [42]. Due to these limitations, researchers often use proxies to measure work-related well-being such as job satisfaction, worker engagement, and mental health status [43,44,45]. The Thriving from Work Questionnaire aims to overcome these limitations by capturing how work and its specific conditions and policies shape the extent to which workers thrive from their work. Through our conceptualization of thriving from work, we recognize that thriving is the result of a bidirectional relationship between an individual and the environments (including work) in which they function.

This paper is important for several reasons. Well-designed instruments are the foundation of many of the variables we measure in our research and practice. Researchers, practitioners and policy-makers need to be confident in the validity of the instruments they use. Boateng et al. in their paper, “best practices for developing and validating scales for health, social and behavioral research” advocate for scale developers to be transparent in reporting their development methods to facilitate the advancement and understanding of the outcome measures we use [46]. Morgado et al. in their systematic review of scale development practices stated that item generation is possibly the most important step of the scale development process, as identification and early testing of these items for face validity is important to ensure the construct is adequately being captured and measured appropriately [47]. Failing to adequality define the conceptual domain of a construct can lead to various problems as a result of poor construct definition [47,48,49]. Thus, the purpose of this paper is to describe the conceptualization and preliminary stages of development of a novel measure of “Thriving from Work”.

## 2. Materials and Methods

We conducted formative research to develop candidate items for the *Thriving from Work Questionnaire* with the following aims:

- to define “thriving from work”
- to identify attributes that could be mapped onto key dimensions of thriving from work
- to develop a comprehensive list of cognitively tested items for later psychometric evaluation and ultimately use in occupational health and safety [OHS] research and practice.

We used an evidence-based multi-step process consisting of several steps: (a) review of the literature on work-related well-being and thriving, and identification of existing measures and related constructs, (b) review of candidate questions resulting from the literature review by the investigator team, (c) workshop to obtain input on a preliminary draft of the questionnaire, and conceptualization and definition of thriving from work, (d) content expert interviews and in-depth review of questionnaire, and, (e) four rounds of cognitive testing with a sample of workers from various sectors and with different demographic characteristics.

### 2.1. Workshop

In December 2019, the Harvard T.H. Chan School of Public Health Center for Work, Health, and Well-being (the Center) sponsored an interactive 90-minute workshop attended by 33 Center members and affiliates from diverse disciplines including epidemiology, biostatistics, occupational and environmental health, law, economics, medicine, psychology, sociology, business, and public health. The main objective of the workshop was to obtain input on a preliminary draft of a questionnaire, and conceptualization and definition of thriving from work, informed by a prior review of the literature. Participants attended in person or by videoconference. In advance of the workshop, we distributed a draft definition and discussion of the concept of thriving from work, based on an initial literature review, as well as candidate items under consideration for use in the Thriving from Work Questionnaire. During the workshop, we obtained feedback and discussed challenges that the investigative team were facing in the development of the questionnaire.

### 2.2. Content Expert Interviews and Review

The study, as originally designed included an in-person modified Delphi process to be conducted in 2020, intended to reach a reasonable level of consensus in the content of the Thriving from Work Questionnaire. The plans for an in-person meeting were revised due to the COVID-19 pandemic. Instead, eighteen content experts from different disciplines and backgrounds were invited to participate individually in a one-time in-person videoconference interview using Voice over Internet Protocol (VoIP) mediated technology (Zoom Video Communications, San Jose, CA, USA). Interviews occurred between May and July 2020: fifteen were conducted jointly by lead investigators (GRW and SP) and three were conducted by one investigator (SP). VOiP is considered acceptable as a replicable method for in-person interviews, as it allows for real-time interaction involving sound, video and even written text using the chat function (this included relevant documents or publications being shared by the experts during the interview) [50]. Interviews were audio-recorded and transcribed using automated features. Interview transcripts were reviewed and summarized after each interview was completed. The lead investigators reviewed and discussed the key findings from each interview and adjusted the questionnaire after each interview, taking into consideration the totality of the previously obtained advice, information, and opinions. Thus, each expert reviewed a potentially different version of the questionnaire. In some instances, the investigators provided questions and options regarding potential items and attributes based on findings from a previous interview’s findings.

The last four interviews provided little new information and were primarily confirmatory in their nature; interviews were stopped at a point where saturation naturally occurred during the data collection.

Experts often suggested a specific attribute of Thriving from Work that they perceived as being very important that they felt had not yet been incorporated into the instrument they reviewed. In these instances, the investigators would first attempt to identify an item from an existing published peer-reviewed questionnaire. If an acceptable item could not be found, the investigator team developed or modified an item and presented both the attribute and item in subsequent expert interviews. Both positive and negative items were identified for some attributes because directionality was considered important by the experts and the investigator team. After the initial round of expert interviews were completed, each expert was asked to participate in a second review of the questionnaire that had been modified based on the initial interviews. The second review was completed electronically via email in late September 2020.

### 2.3. Cognitive Testing with Workers

We conducted a total of four rounds of cognitive testing to ensure that items included on the questionnaire were understood consistently, as intended by the investigators, and to identify and remedy any problems [49]. Following the workshop but prior to the expert review, we conducted a first round of cognitive testing with a convenience sample of six workers. We then tested a revised list of candidate items, that also included items added through the expert panel, through an additional three rounds of cognitive testing with a convenience sample consisting of a total of 20 workers (6–8 participants per round). To the extent possible, workers were purposively recruited from different industries, job levels, education levels, work arrangements, genders, races and ethnicities, and age groups.

We used the think-aloud method with retrospective probing to ask additional clarifying questions on comprehension, information retrieval, judgment/estimation, and selection of response category [48,49]. The interview had two main parts: general questions focused on topics of interest related to the questionnaire, and specific questions regarding items in the questionnaire. General questions focused on the worker’s perception of the meaning of thriving from work, differences between “work” and “job”, questionnaire instructions, response categories and layout. Specific questions asked workers about their interpretation of specific words that the researchers thought might be problematic (for example, “what does realize my full potential” mean to you?”). For some items, the workers were asked, “how would you ask this in your own words” to assess alignment between question intent and understanding. We also asked participants to give examples of some of the more general items, for example “what does the word ‘resources’ mean to you in the statement—I can easily access the resources to do my job well.” We also asked participants to indicate how well each item resonated in relation to their own sense of thriving.

Interviews were conducted either using VOiP, or by phone for workers who were not familiar with or did not have access to video-conference facilities. Interviews were audio-recorded only, and comments regarding each item were summarized and discussed by the investigator team. Substantive changes were made to items between each round.

## 3. Results

### 3.1. Defining Thriving from Work

Our initial investigation from the literature, workshop and expert interviews resulted in our adopting the following definition of Thriving from Work:

*“Thriving from work” is defined as the state of positive mental, physical, and social functioning in which workers’ experiences of their work and working conditions enable them to thrive in their overall lives, contributing to their ability to achieve their full potential in their work, home, and community.*

This definition recognizes that thriving reflects a multidimensional relationship between an individual worker and the environments (including the work environment) in which the individual functions. An individual’s thriving may be increased or diminished because of specific working conditions, or workplace policies and practices. The experiences of a worker at home or in their community is likely to reflect specific working conditions, but the worker’s contribution to their workplace and their perception of the conditions and policies they face at work may also reflect, in part, the other kinds of social, physical, and economic conditions they experience outside of the workplace. For example, workplace schedule predictability and access to leave with short notice may be important in controlling the stresses resulting from being a caregiver. Conversely, the responsibilities and stresses of being a caregiver may influence an individual’s engagement in their work.

### 3.2. Conceptualizing Thriving from Work

Based on an extensive literature review, a draft definition and conceptualization of Thriving from Work was presented at the Center’s workshop. We initially conceptualized Thriving from Work into seven dimensions (Table 1, Column 1) and 13 attributes (Table 1, Column 2). Then, based on the findings from the workshop and the expert review, we added fourteen attributes (Table 1, Column 3). This resulted in a total of 37 attributes mapped to the seven dimensions.

Additional items were generated as a result of both the workshop and expert review process. Source items were either identified through known published reliable and valid instruments, [51,52,53,54,55,56,57,58,59,60] or were developed by the investigator team if not known items could be identified.

### 3.3. Findings from the Cognitive Testing

Four rounds of cognitive testing with a total of 26 workers were conducted (Table 2).

Cognitive testing respondents were provided with the following instructions for the questionnaire: “The following items relate to how you perceive the work you do day-to-day. If you have more than one job, please consider your current job that is most IMPORTANT to you when responding. Please think about this same job when you are answering all of the questions. Indicate how often, if at all, you have generally felt that way about your work over the last month. Select one response for each item.” This introduction was easily understood, and no changes were made.

Every candidate item on the questionnaire was rated on a Likert scale: Always, Almost Always, Usually, Sometimes, Rarely, or Never. ‘Not Applicable’ was also provided as a response option. The ‘Almost Always’ response was added after the second round of cognitive testing following consistent feedback from participants suggesting the need for this category. All other categories were unchanged from the first round of cognitive testing.

Changes to the candidate items were made based on input from multiple respondents and after discussion among the investigators. Modifications were made through each round of the cognitive testing as indicated in Appendix A (Table A1). Some items were dropped because they were too difficult to answer or were identified as redundant. During this process, additional items were added in response to the expert interviews and the results from earlier rounds of cognitive testing. Specific attention was made to the interpretation of items and concepts regarding “work” versus “job” to ensure participants were interpreting items in a similar way. Throughout the cognitive testing process, we found several items to have ambiguous terminology resulting in an unintended restrictive interpretation of an item, frequent responses outside our thriving from work framework, or phrasing that was too complex to be answered within the response categories. Of note, many items that had been taken from previously validated scales needed to be modified for our instrument, first to capture our conceptualization of “thriving from work” and, second, based on our cognitive testing results. The cognitive testing process resulted in a final list of 87 candidate items for the Thriving from Work Questionnaire (Table 3).

## 4. Discussion

The construct, “Thriving from Work”, is a useful, integrated indicator of worker well-being. We describe the process we followed to develop the candidate items for an instrument to measure Thriving from Work, intended to assess the conditions and experiences at work and out-of-work factors that interact with one another and collectively influence a worker’s well-being.

In the context of questionnaire development, response error due to respondent’s poor interpretability of the items, responses, and instructions, can be addressed through methods such as substantive content expert review and cognitive testing [61]. The candidate items yielded through this research have established good face validity and cognitive response properties as single items through rigorous review by substantive content experts and cognitive testing with workers, respectively. Although our intention is to conduct item reduction and psychometric evaluation on the instrument through future studies, researchers can use individual items from the current questionnaire and interpret them directly.

Positive health outcomes that result from workplace policies, practices, and exposures are often overlooked in the OHS scientific literature that focuses more commonly on identifying risks of injury or ill-health. Current challenges, such as the COVID-19 pandemic, have called attention to the importance of embracing a well-being framework as workers return to work and continue to struggle with the mental and physical sequalae of the pandemic [62,63,64]. NIOSH, as well as other international organizations, have emphasized the value of incorporating a positive well-being focus.

NIOSH recently published a framework of worker well-being [24], and an integrated assessment of worker well-being (WellBQ) [38], based on a substantive literature review and expert panel. The panel concluded that worker well-being is a multi-dimensional, dynamic, and integrative concept. They defined worker well-being as the “experience of positive perceptions, and the presence of constructive conditions at work and beyond that enables workers to thrive and achieve their full potential” [24] (p. 592). This definition captures well-being as a continuum, with thriving considered as the highest level of well-being. Our conceptualization and instrument complement the NIOSH WellBQ. Both have a focus on the relationship of working policies, practices, and conditions as well as conditions outside of work that are influenced by and influence the work experience, and recognize well-being as a comprehensive, multi-dimensional integrated measure.

The Thriving from Work Questionnaire is intended to serve multiple purposes across research, policy, and practice. Next steps in the development of the Thriving from Work Questionnaire include item reduction and psychometric evaluation of the instrument across samples of diverse workers, as well as design of a scoring and benchmarking system. We have designed a series of studies to validate our questionnaire, and systematically reduce the number of items, in an online sample of U.S. workers, as well as in various industry-specific samples. Although this process will lead to a briefer instrument, it is important to note that the 87 questions identified through this research provide a battery of cognitively tested items that are ready for current use (Table 3).

Of note, although we used an international expert panel, the questionnaire was cognitively tested with U.S. workers and therefore items may not be as useful without modification in countries outside of the U.S. where labor, economic and social security systems differ. In these settings, additional cognitive testing with workers is recommended. Additionally, we modified our original methods due to the COVID-19 pandemic. For example, we had planned to conduct the expert panel as an in-person one-day workshop and instead converted to videoconference interviews. The value of having experts discuss the topics and the questionnaire as a group might have yielded different findings; however, the benefit of conducting interviews virtually meant we could expand to an international panel. Additionally, while there have been reported concerns regarding the technological issues that may be faced conducting interviews by video conference [50], we did not experience any such problems.

## 5. Conclusions

The importance of work-related well-being, and thriving from work, as critical worker outcomes are supported by both evidence and practice. Therefore, evidence-based practical, reliable, and validated measures are needed to measure these positive health outcomes. This research will serve as the foundation for the Thriving from Work Questionnaire which is being developed to meet this need. Through our formative research, our goal was to develop a battery of cognitively tested candidate items that will have broad utility across workers from different occupations and industries. The questionnaire will have application for assessing the consequences of workplace modifications and interventions, including those specifically designed to improve worker well-being. We were able to demonstrate that the candidate items for the Thriving from Work Questionnaire effectively assessed the defined attributes of Thriving from Work based on our definition and conceptualization across seven broad dimensions. Items were established as having good face validity and cognitive response properties; researchers and practitioners may consider the individual questionnaire items in their own survey research. Future research will focus on item reduction and psychometric evaluation of the questionnaire.

Thriving from Work is an important positive health and well-being construct, that measures the extent an individual thrives because of their working conditions and experience of work, not only at work but also in their life beyond work. Future research is needed to identify how work can have positive health and well-being impacts to create thriving workforces and workplaces, in which workers thrive not only at work but in their lives outside of work. As future work continues to change and evolve, future research should also focus on examining how workers’ thriving–its definition and conceptualization–may also be evolving.

## Figures and Tables

**Table 1:** Thriving from Work: Dimensions and Attributes.

Dimensions of Thriving from Work	Initial Attributes Drafted from the Literature Review	Attributes Added Through the Workshop and Expert Review
*Psychological well-being from work*	Meaning & Purpose Growth & development	Values align with company
*Emotional well-being from work*	Job satisfaction Happiness Engagement	Enthusiasm Contribution to life satisfaction
*Social well-being from Work*	Supportive relationships Valued Belonging Respect Contributions to others	Fair treatment Worker voice Recognition
*Work–life integration*	Work–life balance Commuting	Work-Family Job creep
*Basic needs for Thriving from Work*	Job security Pay	Benefits Opportunities for promotion
*Job design and experience of work*	Autonomy Schedule control Job demands Physical work environment Adequate resources Skills & knowledge	Work intensity
*Health, and Physical and Mental Well-being from Work*	Physical Safety Psychological Safety Energy	Stress Injury Exhaustion

**Table 2:** Details of Cognitive Testing Participants (*n* = 26).

		Mean (SD; Range)
**Age (Years)**		36.2 (10.6; 24 to 69)
		**% (*n*)**
Gender	Male	62% (16)
	Female	38% (10)
Race/Ethnicity	White	46% (12)
	Asian	27% (7)
	Hispanic/Latinx	15% (4)
	Black	8% (2)
	Native American	(1)
Main language spoken at home	English	85% (22)
	Other	15% (4)
Education Level	Completed Grade 12/High school	15% (4)
	Some college or technical school	23% (6)
	College degree	42% (11)
	Graduate/Master’s degree	20% (5)
Main Occupation	Hospitality/Food service	19% (5)
	Construction	12% (3)
	Education	12% (3)
	Healthcare	12% (3)
	Transportation	12% (3)
	Military/security	8% (2)
	Professional Services (e.g., human resources, media relations)	8% (2)
	Agriculture/farming	(1)
	Entertainment	(1)
	Retail	(1)
	Manufacturing	(1)
	Information Technology	(1)
	Technical/Scientific	(1)
Number of jobs/employers	One	88% (23)
	Two or more	12% (3)
Employment status	Full time, salaried	54% (14)
	Full time, shift work with overtime	8% (2)
	Part time/hourly	23% (6)
	Contract	8% (2)
	App-based	(1)
	Work without pay (e.g., family farm)	(1)

**Table 3:** Thriving from Work Questionnaire: Final Candidate Questions.

Dimension	Item
*Psychological* *Well-being* *from Work*	My work gives me a sense of purpose.
	My work adds meaning to my life.
	My work makes a meaningful contribution to society.
	My work allows me to develop new knowledge and skills.
	My job allows me to achieve my full potential.
	At work, I have the opportunity to do what I do best every day.
	The things I am asked to do at work are consistent with my personal values.
	I get asked to do things at work that I don’t feel comfortable doing.
*Emotional* *Well-being* *from Work*	I feel engaged by my work.
	At work, my mind is focused on my job.
	I am satisfied with my job.
	I am satisfied with the kind of work I do.
	The kind of work I do makes me happy.
	My job makes me happy.
	My work adds to my overall life satisfaction.
	I love my job.
	I am enthusiastic about my work.
	My job is pointless (has no useful purpose).
	My job is boring.
*Social* *Well-being from Work*	I receive recognition at work for my accomplishments
	I feel supported by the people I work with.
	I feel supported by my coworkers.
	I feel supported by my managers/supervisors.
	I receive useful and timely feedback at work from my managers/supervisors.
	I feel valued by the people I work with.
	I feel valued by my coworkers.
	I feel valued by my managers/supervisors.
	I feel valued by other people I interact with at work, such as customers, clients, students, patients (any other people who are NOT your supervisors or coworkers).
	My work is valued by others.
	My work is valued by my coworkers.
	My work is valued by my managers/supervisors.
	My work is valued by other people I interact with at work, such as customers, clients, students, patients (any other people who are NOT your supervisors or coworkers).
	At work, I feel like I belong.
	I am comfortable being myself at work.
	I am treated with respect at work.
	I am treated with respect by my coworkers.
	I am treated with respect by my managers/supervisors
	I am treated with respect by other people I interact with at work, such as customers, clients, students, patients (any other people who are NOT your supervisors or coworkers).
	I am treated fairly at work.
	I am treated fairly by my coworkers.
	I am treated fairly by my managers/supervisors.
	I am treated fairly by other people I interact with at work, such as customers, clients, students, patients (any other people who are NOT your supervisors or coworkers).
	I am bullied, harassed, or humiliated at work.
	My work allows me to contribute to the happiness and well-being of others.
	I can voice concerns or make suggestions at work without getting into trouble.
	At work, my opinions matter.
	No one cares about my opinions at work.
*Work–life* *Integration*	I can easily manage my job as well as attend to my needs and the needs of my family.
	I can achieve a healthy balance between my work and my life outside of work.
	I worry about things at work when I am not working.
	My family and friends value the work I do.
	Travelling to and from work is stressful for me.
	Travelling to and from work is easy and stress-free.
	I feel safe getting to and from work.
*Basic Needs for Thriving from Work*	I feel my job is secure.
	I am grateful for my job.
	I am paid fairly for the job I do.
	My pay meets my needs and the needs of my family.
	I am not paid enough money to make ends meet.
	I am satisfied with the employee benefits provided through my work, such as access to health insurance, life insurance, a pension or retirement savings plan.
	I am satisfied with the amount of paid vacation days I get.
	I am satisfied with the amount of paid leave I can take to care for myself or family members.
	I have good opportunities for promotion.
*Job Design and Experience of Work*	I can easily manage the demands of my job.
	I have more work to do than I can complete during paid work hours.
	I am happy with how much input I have in decisions that affect my work.
	I have control over how my daily work is done.
	I can solve problems at work without having to ask for permission.
	I am happy with how much control I have over my work schedule.
	I can schedule a day off or take vacation when I want or need to.
	I can take unpaid leave if I need to.
	I have enough time, within my normal working hours, to get my job done.
	I have adequate control over the pace of my work.
	My physical work environment (workspace, light, temperature) is set up in a way that helps me do my job well.
	Noise at work interferes with my ability to get the job done.
	I have the skills and knowledge I need to do my job well.
	I have access to the resources I need to do my job well.
	I know enough about what is going on in my company/organization to do my job well.
*Health, and Physical and Mental Well-being from Work*	I feel physically safe at work.
	I feel psychologically safe at work.
	After I leave work, I have enough energy to do the things I want or need to do.
	I am too tired after work to enjoy things.
	I feel excessive levels of stress at work.
	I worry that I will get hurt at work.
	I find work emotionally exhausting.
	I find work physically exhausting.
	My work contributes in a positive way to my well-being.

## Appendix A

**Table A1:** Findings from the cognitive testing of Thriving from Work Questionnaire candidate items.

Attribute	Round 1	Round 2	Round 3	Round 4
**Psychological Well-Being from Work**				
*Purpose*	My work gives me a sense of purpose.	*No change.*	*No change*	*No change.*
*Meaning*	My work gives meaning to my life.	*No change.*	My work adds meaning to my life. ^c^	*No change*
				My work makes a meaningful contribution to society. ^b^
*Growth and Development*	My work allows me to develop new knowledge and skills.	*No change.*	*No change.*	*No change.*
	My work supports my interests.	*No change.*	*No change.*	*DROPPED ^b^*
		I feel that my job allows me to realize my full potential. ^a^	My job allows me to realize my full potential. ^b^	My job allows me to achieve my full Potential. ^b^
				At work, I have the opportunity to do what I do best every day. ^b^
*Values Align with Company*			The things I am asked to do at work are consistent with my personal values. ^a^	*No change.*
			I get asked to do things at work that I don’t feel comfortable doing.	*No change.*
**Emotional Well-being from Work**				
*Engagement*	I am engaged in my work.	*No change.*	I feel engaged by my work. ^b^	*No change.*
				At work, my mind is focused on my job. ^b^
*Job Satisfaction*	Overall, I am satisfied with my job.	*No change.*	*No change.*	I am satisfied with my job. ^b^
	Overall, I am satisfied with the work I am doing.	*No change.*	Overall, I am satisfied with the work I do. ^b^	I am satisfied with the kind of work I do. ^b^
*Happiness*	My work makes me happy.	My work makes me happy. ^d^	*No change.*	The kind of work I do makes me happy. ^b^
		My job makes me happy. ^d^	*No change.*	*No change.*
	I look forward to going to work each day.	DROPPED ^c^		
*Contribution to Life Satisfaction*			My work makes a positive contribution to my overall life satisfaction. ^a^	My work adds to my overall life satisfaction. ^b^
*Enthusiasm*			I love my job. ^a,b,d^	*No change.*
			I am enthusiastic about my work. ^a^	*No change.*
			My job is pointless (has no useful purpose). ^d^	*No change.*
			My job is boring. ^d^	*No change.*
**Social Well-being from Work**				
*Supportive Work Relationships*				I feel supported by the people I work with.
	I have a supportive relationship with my coworkers.	*No change.*	I feel supported by my coworkers.	*No change.*
	I have a supportive relationship with my supervisor.	I feel supported by my supervisor.	*No change.*	I feel supported by my managers/supervisors.
			I receive useful and timely feedback at work from my supervisors. ^a^	I receive useful and timely feedback at work from my managers/supervisors. ^b^
			I feel supported by my employer (e.g., workplace policies).^a^	DROPPED ^b^
*Valued at Work*	I am valued at work by my colleagues and supervisors.	*No change.*		I feel valued by the people I work with.
				I feel valued by other people I interact with at work, such as customers, clients, students, patients (any other people who are NOT your supervisors or coworkers).^b^
			I feel valued by my coworkers. ^b^	*No change.*
			I feel valued by my supervisors. ^b^	*No change.*
				My work is valued by others. ^b^
		The work that I do is valued by my colleagues and Supervisors. ^a^	My work is valued by my coworkers. ^b^	*No change.*
				My work is valued by other people I interact with at work, such as customers, clients, students, patients (any other people who are NOT your supervisors or coworkers).^b^
			My work is valued by my supervisors. ^b^	*No change.*
*Belonging in the Workplace*		I feel like I am an important part of a team or community at my workplace. ^a^	I feel like I am part of a community at work. ^b^	DROPPED ^b^
		I feel like I belong in my workplace. ^a^	At work, I feel like I belong. ^b^	*No change.*
		I am comfortable being myself at work. ^a^	*No change.*	*No change.*
*Respect*	I am treated with respect in the workplace.	*No change.*	*No change.*	I am treated with respect at work. ^b^
				I am treated with respect by my coworkers. ^b^
				I am treated with respect by my managers/supervisors. ^b^
				I am treated with respect by other people I interact with at work, such as customers, clients, students, patients (any other people who are NOT your supervisors or coworkers).^b^
*Fair Treatment*			I am treated fairly at work ^a^	I am treated fairly at work. ^b^
				I am treated fairly by my coworkers. ^b^
				I am treated fairly by my managers/supervisors ^b^
				I am treated fairly by other people I interact with at work, such as customers, clients, students, patients (any other people who are NOT your supervisors or coworkers).^b^
				I am bullied, harassed, or humiliated at work ^d^
*Contributions to Others*	My work allows me to contribute to the happiness and wellbeing of others.	*No change.*	*No change.*	*No change.*
*Worker Voice*		I can voice concerns or make suggestions at work without getting into trouble. ^d^	*No change.*	*No change.*
			At work, my opinions matter. ^a^	*No change.*
				No one cares about my opinions at work. ^b^
*Recognition*				I receive recognition at work for my Accomplishments. ^d^
**Work-Life Integration**				
*Work-Life Balance*	I am satisfied with my work-life balance.	*No change.*	I am able to achieve a healthy balance between my work and my life outside of work. ^b^	I can achieve a healthy balance between my work and my life outside of work. ^b^
*Work-Family*	The demands of my work interfere with my family or personal time.	DROPPED ^c^		
	The demands of my work make it difficult to fulfil my family or personal responsibilities/duties.	DROPPED ^c^		
	I can easily manage my job as well as attend to the needs of myself or my family.	*No change.*	I can easily manage my job as well as attend to my needs and the needs of my family. ^b^	*No change.*
				My family and friends value the work I do. ^d^
*Job Creep*			I worry about work problems when I am not working. ^d^	I worry about things at work when I am not working. ^b^
*Commuting*	My commute to work in not a problem for me.	My commute to work is a problem for me. ^b^	My commute to work is safe, easy and stress-free. ^b^	Traveling to and from work is easy and stress-free. ^b^
			Travelling to and from work is a problem for me. ^b^	Travelling to and from work is stressful for me. ^b^
			I feel safe getting to and from work. ^b^	*No change.*
*Opportunities for Promotion*				I have good opportunities for promotion. ^b,d^
**Basic Needs for Thriving from Work**				
*Job Security*	I feel my job is secure.	*No change.*	*No change.*	*No change.*
				I am grateful for my job.
*Pay*	I am paid fairly for the job I do.	*No change.*	*No change.*	*No change.*
			I receive adequate pay to meet my needs. ^a^	My pay meets my needs and the needs of my family. ^b^
				I am not paid enough money to make ends meet. ^b^
*Benefits*		I am satisfied with the employee benefits (e.g., health insurance, retirement fund, time off, wellness initiatives) that are provided through my work. ^a^	*No change.*	I am satisfied with the employee benefits provided through my work, such as access to health insurance, life insurance, a pension or retirement savings plan. ^b^
				I am satisfied with the amount of paid vacation days I get. ^b^
				I am satisfied with the amount of paid leave I can take to care for myself or family members. ^b^
**Job Design and Experience of Work**				
*Job Demands (General)*	I can easily manage the demands of my job.	*No change.*	*No change.*	*No change.*
	I spend more time than I would like to complete unpaid work-related activities (e.g., work travel, waiting for my next job, completing work tasks on my own time).	*No change.*	*No change.*	DROPPED ^b^
			I have more work to do than I can complete during paid work hours. ^d,b^	*No change.*
*Autonomy*	I am satisfied with how much say I have in decisions that affect my work.	*No change.*	I am happy with how much say I have in decisions that affect my work. ^b^	I am happy with how much input I have in decisions that affect my work. ^b^
				I have control over how my daily work is done. ^d^
			I can solve problems at work without having to ask for permission. ^a^	*No change.*
*Schedule Control*	I am happy with how much control I have over my work schedule.	*No change.*	*No change.*	*No change.*
			I can schedule a day off work when I want or need to. ^a,d^	*No change.*
			I can take unpaid leave when I need to. ^a,d^	
			I have no control over my work hours. ^a,d^	
*Work Intensity*			I have enough time, within my normal working hours, to get my job done. ^a,d^	*No change.*
			I have no control over the pace of my work. ^b,d^	DROPPED ^b^
			I have adequate control over the pace of my work. ^b,d^	*No change.*
*Working Conditions (General)*	My working conditions contribute in a positive way to my health and well-being.	*No change.*	*No change.*	My work contributes in a positive way to my health and well-being. ^b,d^
*Physical Work Environment*	The space I work in is conducive to getting my work done.	*No change.*	*No change.*	My physical work environment (workplace, light, temperature) is set up in a way that helps me to get my work done in the best way possible. ^b^
			Noise at work interferes with my ability to get the job done. ^d^	*No change.*
*Skills and Knowledge*	I have the skills and knowledge to do my job well.	I have the skills and knowledge I need to do my job well. ^b^	*No change.*	*No change.*
*Adequate Resources*	I can easily access the resources to do my job well.	*No change.*	I have the resources to do my job well. ^b^	I have access to the resources I need to do my job well. ^b,d,e^
	I know enough about what is going on in my organization to do my job well.	*No change.*	*No change.*	I know enough about what is going on in my company/organization to do my job well. ^b^
**Health, and Physical and Mental Well-being from Work**				
*Physical Safety*	I feel physically safe at work.	*No change.*	*No change.*	*No change.*
*Psychological Safety*	I feel psychologically safe at work.	*No change.*	*No change.*	*No change.*
*Energy*	When I come home from work, I have enough energy to do the things I want or need to do.	After I finish work, I have enough energy to do the things I want or need to do. ^b^	After I leave work, I have enough energy to do the things I want or need to do. ^b^	*No change.*
			I am too tired after work to enjoy things. ^d^	*No change.*
*Stress*			I feel excessive levels of stress at work. ^d^	*No change.*
*Injury*			I worry that I will get hurt at work. ^d^	*No change.*
*Exhaustion*			I find work emotionally exhausting. ^d^	*No change.*
			I find work physically exhausting. ^d^	*No change.*

a- Added through either workshop or expert review. b- Changes made based on findings from cognitive interviews and input from investigator team. c- Item dropped as performed poorly through this round of cognitive testing. d- Additions made by the investigator team based on current evidence and/or investigator expertise. e- After this item was modified it was moved to the “Health, & physical and mental well-being domain”.
